# Leadership, politics, and communication: challenges of the epidemiology workforce during emergency response

**DOI:** 10.1186/s12960-022-00727-y

**Published:** 2022-04-11

**Authors:** Amy Elizabeth Parry, Martyn D. Kirk, Samantha Colquhoun, David N. Durrheim, Tambri Housen

**Affiliations:** 1 National Centre for Epidemiology & Population Health (NCEPH), Acton, ACT, The Australian National University (ANU), Building 62 Mills Road, Canberra, Australia; 2 University of Newcastle, Newcastle, NSW Australia

**Keywords:** Epidemiology, Public health practice, Health workforce, Emergency, Leadership, Communication

## Abstract

**Background:**

Improving the epidemiological response to emergencies requires an understanding of who the responders are, their role and skills, and the challenges they face during responses. In this paper, we explore the role of the epidemiologist and identify challenges they face during emergency response.

**Methods:**

We conducted a cross-sectional survey to learn more about epidemiologists who respond to public health emergencies. The online survey included open and closed-ended questions on challenges faced while responding, the roles of epidemiology responders, self-rating of skills, and support needed and received. We used purposive sampling to identify participants and a snowballing approach thereafter. We compared data by a number of characteristics, including national or international responder on their last response prior to the survey. We analysed the data using descriptive, content, and exploratory factor analysis.

**Results:**

We received 166 responses from individuals with experience in emergency response. The most frequently reported challenge was navigating the political dynamics of a response, which was more common for international responders than national. National responders experienced fewer challenges related to culture, language, and communication. Epidemiology responders reported a lack of response role clarity, limited knowledge sharing, and communication issues during emergency response. Sixty-seven percent of participants reported they needed support to do their job well; males who requested support were statistically more likely to receive it than females who asked.

**Conclusions:**

Our study identified that national responders have additional strengths, such as better understanding of the local political environment, language, and culture, which may in turn support identification of local needs and priorities. Although this research was conducted prior to the COVID-19 pandemic, the results are even more relevant now. This research builds on emerging evidence on how to strengthen public health emergency response and provides a platform to begin a global conversation to address operational issues and the role of the international epidemiology responder.

## Background

The global public health emergency response workforce deploy to control infectious disease outbreaks as well as respond to disasters to keep communities safe. To ensure that the public health response to events of international and national concern is timely and effective, we need to optimise workforce performance quality and impact [1–3].

Health workforce development is a fundamental activity required to meet the obligations of the International Health Regulations (IHR 2005) [4]. The Joint External Evaluation (JEE) is a tool aimed to identify how countries are tracking towards meeting these obligations. Findings from the JEE highlight that the public health workforce needs strengthening to effectively manage health security events [5–8]. In many countries, field epidemiology training programs (FETP) are implemented as a key activity to strengthen technical expertise within the existing workforce and to meet the target of 1 epidemiologist per 200,000 persons [9–12].

There is limited evidence on emergency response workforce best practice and there is a need for stronger scientific evidence and innovative research and evaluation methods [13–16]. The reasons for this are unclear but may relate to the lack of operational plan testing and poorly defined job roles. Epidemiology is often not included in national health workforce plans and the role may have inconsistent skill and knowledge requirements [16]. In recent years, role formalisation of other emergency response health professions has taken place, however, not for epidemiology [17]. The lack of clarity on the role of the epidemiologist has been identified in previous research as a primary barrier to effective emergency response [2, 16, 18].

The COVID-19 pandemic has demonstrated the importance and value of the public health workforce, and the requirement for specialist epidemiology response skills and expertise. The Independent Panel’s COVID-19 pandemic report and the 2021 World Health Assembly identified critical failures in responding to the COVID-19 crisis, including the under-resourced health workforce [19–21]. It is clear that investing in and strengthening the public health emergency response workforce is an international priority. For well-targeted investment in workforce strengthening, we need to understand the challenges and barriers faced during public health emergency response.

International epidemiology emergency response work is most commonly short-term, which has led to a cycle of reaction during emergencies with limited or no documentation of the lessons learned, or modification of future response actions. After action reviews, or response evaluations, will go some way to addressing this, however, they are an under-utilised tool [22]. For a stronger, better-managed epidemiology response, it is important to understand who the responders are, their role and skills, as well as how skills and knowledge are shared. In this paper, we explore the role of the epidemiologist and identify challenges they face during emergency response.

## Method

We conducted a cross-sectional survey of emergency response epidemiologists. We have previously reported the methods of the survey elsewhere, and describe them here in brief [2, 23].

### Study population

Participants of this survey were epidemiologists who had previously worked in an epidemiology role during an emergency response (Box 1).

### Box 1: Terminology definitions provided to survey respondents

**Table:** 

Emergency response: A public health issue that requires external assistance. This could mean a request for national and/or a request for international support. The crisis may be manmade (such as armed conflict), natural hazard (such as drought, flood) or the result of an infectious disease outbreak Epidemiology role: The role performed by any person who participates in surveillance, response, or disease control activities during an emergency	

### Sampling and recruitment

Purposive sampling was initially used to identify participants, a snowballing method was then used to broaden participation [24, 25].

Recruitment consisted of multiple strategies. We produced a YouTube video to explain the purpose of the study; this video was produced in English and included optional multi-language subtitles. We disseminated the video via Twitter, LinkedIn, and Facebook, and delivered eight reminders to participate via these platforms over 3 months. Our international partner, TEPHINET (Training Programs in Epidemiology and Public Health Interventions Network) supported recruitment through conducting a parallel social media campaign. In addition, TEPHINET contacted FETP alumni and FETP country programs via email and through the TEPHINET website.

We advertised the survey at the 10th TEPHINET Global Scientific Conference, the Australian Communicable Diseases Control Conference 2019, and the European Scientific Conference on Applied Infectious Disease Epidemiology (ESCAIDE 2019). Survey respondents were requested to forward the survey link to other applied epidemiologists in their professional network.

### Data collection

Survey questions were based on findings from key informant interviews conducted in 2019 and issues raised in the literature [16, 26]. Questions were reviewed by the key informant interviewees and pre-tested with 11 field epidemiologists with experience in emergency response. The survey was created and tested in English and then translated into French and back-translated to ensure accuracy. The survey was self-administered online by respondents via a REDCap (Research Electronic Data Capture) secure survey between October 2019 to February 2020. No incentives were provided for participation. The survey questionnaire is detailed elsewhere [2].

In this analysis, we examined questionnaire data on (1) challenges faced while responding, (2) epidemiology responder roles, (3) self-rating of skills, and (4) support needed and received. Question format included checkbox items and Likert scales, each section included an optional open-ended question with free-text field, allowing participants to provide further comments. To contextualise answers, we asked respondents to reflect on their most recent emergency response experience. Due to the length of the survey, most questions were not compulsory; therefore, the denominator varies between questions. Questions related to ‘support’ were collected in two categories. ‘Technical’ refers directly to epidemiological support; ‘non-technical’ refers to other support including but not limited to communication, well-being, understanding response context, or administrative.

### Ethics and consent

Participation in this study was voluntary and anonymous. We provided plain language study information in French and English. Each participant provided online consent before accessing the survey questions. During the survey, the participants could remove or change answers before survey submission. Ethics approval was provided by the Australian National University Human Research Ethics Committee (ID:2019-068).

### Data analysis

We conducted descriptive analysis of survey data in Microsoft Excel and Stata15 (TX:StataCorp). We conducted content analysis on answers to open-ended questions; the text was open-coded and common categories were developed iteratively [27, 28].

In our analysis, we compared findings based on whether the participant’s latest response was as an international or national responder (termed in this paper as ‘responder type’). We also compared findings based on identified gender, epidemiology experience, epidemiology emergency response experience, and location. We grouped respondents by geographical areas according to the six World Health Organization (WHO) regions; Africa, Eastern Mediterranean, Europe, Americas, South–East Asia, and the Western Pacific [29].

For questionnaire responses on roles and challenges, we performed an exploratory factor analysis to identify closely related items and distil the large data set into fewer groupings [16, 17]. Eigenvalues and scree plots were reviewed to determine the number of groupings, orthogonal varimax rotation then supported the identification and interpretation of groupings of latent themes [30]. Factor loadings are the correlation between an item and a factor, a factor loading score from 0 to 1 indicates relationship between the item measured in that category [30]. Items were included in the factor if the loading was higher than 0.35 [30]. The items found to contribute to a factor were then included in a scale, where each item was equally weighted. For the scales identified through this factor analysis, Cronbach alpha (α) measured internal consistency between items was calculated to determine the scale reliability for future use. Typically, α is considered reliable at > 0.7, but moderate reliability can be identified at > 0.6 [31–33].

To test for statistical difference, we used Pearson’s chi-square for dichotomous data or two-sample Wilcoxon rank-sum (Mann–Whitney) test for data with multiple answer options. We considered results significant if they fell within the 95% confidence interval.

## Results

### Participant characteristics

We received 166 surveys, with representation from all WHO regions. There was a similar distribution of females and males among survey participants (Table 1). The majority of respondents were under 40 years, with a median age of 39 (range: 23–77 years). A third of respondents reported less than 5 years of epidemiology experience and almost half had participated in three or fewer emergency response events in an epidemiology role (Table 1). Fifty-nine percent (96/162) reported their last response was a national response, while 41% (66/162) reported that it was an international response.

**Table 1:** Demographics and characteristics of epidemiology emergency response survey participants (*n* = 166*), 2019–2020

Category	Items	*n* = 166 (100%)
Survey language	English	157 (95%)
	French	9 (5%)
Age (years)	< 20	0 (0%)
	20–29	10 (6%)
	30–39	78 (47%)
	40–49	47 (28%)
	50–59	25 (15%)
	60 +	6 (4%)
Identified gender	Female	85 (51%)
	Male	79 (48%)
	Not reported	2 (1%)
Region (WHO region)	African	43 (26%)
	Eastern Mediterranean	6 (4%)
	Europe	20 (12%)
	Americas	47 (28%)
	South–east Asia	14 (9%)
	Western Pacific	36 (22%)
Responder type (*n* = 162)	National	96 (59%)
	International	66 (41%)
Training type	FETP	129 (78%)
	Non-FETP	37 (22%)
Epidemiology experience (*n* = 164)	< 5 years	53 (32%)
	5 + years	113 (68%)
Any emergency response experience	National ≤ 5 events (*n* = 127)	82 (65%)
	International ≤ 5 events (*n* = 84)	64 (76%)
Epidemiology emergency response experience (*n* = 164)	≤ 3 events	79 (48%)
	4 + events	85 (52%)
Emergency type experience**	Natural disaster	70 (42%)
	Pandemic response	50 (30%)
	Infectious disease outbreak response	146 (88%)
	Conflict	32 (20%)
	Refugee/displaced persons	53 (32%)
	Nutrition emergency	21 (13%)
	Other man-made disasters	12 (7%)
	Other	9 (5%)
Terms of Reference (ToR)	Had ToR (*n* = 163)	78 (44%)
	ToR matched work (*n* = 78)	48 (61%)
Contract length (*n* = 163)	Less than 1 month	52 (32%)
	1 to < 2 months	37 (23%)
	2 to < 3 months	18 (11%)
	3 to < 6 months	20 (12%)
	6 to < 12 months	21 (13%)
	12 + months	14 (9%)

*Denominator was 166 unless otherwise stated

**Includes multiple answers per respondent

### Factor analysis

We asked respondents to identify challenges they had experienced during their most recent emergency response (Table 2). Of the 18 listed challenges in the survey, exploratory factor analysis identified five factors with an eigenvalue over 1 [30]. After scree plot review we reduced five contributing factors to four; ‘*communication’, ‘culture and relationships’, ‘technical skills’*, and ‘*political dynamics and security’* [34]. One item, ‘*language*’, was not statistically related to other challenges, therefore, was analysed as a standalone item (Table 2). The Cronbach alpha for each of these scales was moderately reliable (> 0.6) with the exception of ‘*political dynamics and security*’, indicating the series of questions could be useful in future analysis (Table 2).

**Table 2:** Frequency of reported challenge groupings by epidemiology emergency response survey participants (*n* = 166), 2019–2020

Grouping	*n* = 166 (%)	Eigen value	Cronbach *α*	Itemised challenge	Factor loading	*n* = 166 (%)	Responder type * (*p*)	Item-total correlation
1.Communication (*n* = 7 challenges)	76 (46%)	3.9	0.74	‘Nobody told me what had already been done’	0.70	27 (16%)	0.09	0.68
				‘The team leader did not know how to use me/my skills’	0.68	30 (18%)	0.049	0.65
				‘I did not understand my role’	0.66	19 (11%)	0.26	0.70
				‘The priorities of the response were not communicated to me’	0.63	22 (13%)	0.06	0.69
				‘Nobody told me what to do’	0.61	22 (13%)	0.34	0.68
				‘There were too many other epidemiologists’	0.51	12 (7%)	0.50	0.50
				‘My deployment period was too short to be effective’	0.36	32 (19%)	0.69	0.48
2.Culture and relationships (*n* = 4 challenges)	19 (11%)	2.1	0.66	‘There was nobody I could ask cultural questions to’	0.82	2 (1%)	0.09	0.84
				‘I felt it was difficult to develop relationships with international colleagues’	0.74	6 (4%)	0.003	0.69
				‘I did not understand the culture’	0.60	9 (5%)	0.10	0.84
				‘I felt it was difficult to develop relationships with local colleagues’	0.60	10 (6%)	0.54	0.70
3.Technical skills (*n* = 3 challenges)	24 (14%)	1.6	0.67	‘I did not have the right technical skills needed in the field’	0.75	12 (7%)	0.25	0.81
				‘I found it difficult to apply my skills to the required work’	0.73	11 (7%)	0.74	0.78
				‘I did not have the right software skills’	0.63	15 (9%)	0.09	0.74
4.Political dynamics and security (*n* = 3 challenges)	88 (53%)	1.4	0.52	‘Political dynamics were challenging to understand and work within’	0.73	58 (35%)	0.03	0.71
				‘Security issues affected my capacity to work’	0.64	36 (22%)	0.04	0.71
				‘Collaborating with other partners outside my agency was difficult’	0.53	45 (27%)	0.552	0.72
5. Language (*n* = 1 challenge)	20 (12%)	–	–	‘My language skills were insufficient to need’	0.39	20 (12%)	0.001	–

*Responder type (international or national responder)

The exploratory factor analysis of the ‘epidemiology role’ data condensed 37 items on epidemiology responder activities to nine factors with an eigenvalue of over 1.0 [30]. After scree plot review we reduced the contributing factors to seven factors;[34]. ‘*response guidance*’, *‘data’, ‘investigation’, ‘surveillance’, ‘management’, ‘information’,* and *‘cross-sectoral collaboration’* (Table 3). These categories were not discrete, 5% (9/166) of respondents reported activities in a single category: 17% (29/166) reported activities across all seven categories (Table 3). The Cronbach alpha for each of these scales except for the role of ‘*information’ and ‘cross sectoral collaboration’* were at least moderately reliable (> 0.6) (Table 3).

**Table 3:** Frequency of reported role groupings by epidemiology emergency response survey participants (*n* = 166)*, 2019–2020

Factor grouping	Factor grouping *n* = 166 (%)	Eigen value	Cronbach α	Responder type** *(p)*	Itemised activities	Activities reported *n* = 166(%)	Factor loading	Item-total correlation
Response guidance (*n* = 11 activities)	141 (85%)	9.9	0.84	0.15	Response activity planning	52 (31%)	0.72	0.74
					Sharing information	87 (52%)	0.71	0.71
					Activity prioritisation	47(28%)	0.68	0.74
					Communicating findings	86 (52%)	0.61	0.67
					Response evaluation	43 (26%)	0.60	0.73
					Mentoring	52 (31%)	0.57	0.55
					Report writing	100 (60%)	0.52	0.64
					Needs assessment	45 (27%)	0.47	0.56
					Risk assessment	57 (34%)	0.45	0.55
					Research	42 (31%)	0.41	0.50
					Mapping	41 (25%)	0.41	0.49
Management (*n* = 5 activities)	107 (65%)	1.6	0.69	0.14	Team manager/ supervisor	50 (30%)	0.79	0.73
					Field coordinator	50 (30%)	0.75	0.74
					Epidemiology team lead	62 (37%)	0.46	0.60
					Managing control measures	36 (22%)	0.44	0.70
					Non-epidemiology work	47 (28%)	0.38	0.59
Cross-sectoral collaboration (*n* = 2 activities)	56 (34%)	1.2	0.58	0.004	Source trace-back	20 (12%)	0.67	–
					Collaborating with other sectors	52 (31%)	0.63	–
Data (*n* = 4 activities)	119 (72%)		0.89		Data cleaning	79 (48%)	0.84	0.92
					Data management	83 (50%)	0.83	0.88
					Data analysis	100 (60%)	0.78	0.87
					Data entry	68 (41%)	0.77	0.81
Investigation (*n* = 6 activities)	113 (68%)	2.2	0.85	0.005	Active case finding	61 (37%)	0.81	0.81
					Contact tracing	46 (28%)	0.78	0.75
					Interviews	62 (37%)	0.76	0.81
					Case investigation	72 (43%)	0.74	0.78
					Line listing	60 (36%)	0.63	0.76
					Data collection	82 (49%)	0.46	0.65
Surveillance (*n* = 5 activities)	118 (71%)	2.0	0.81	0.82	Surveillance analysis	84 (51%)	0.79	0.82
					Surveillance monitoring	82 (49%)	0.74	0.73
					Surveillance evaluation	54 (33%)	0.67	0.74
					Surveillance set up	65 (39%)	0.53	0.75
					Development of data collection tools	80 (48%)	0.48	0.74
Information (*n* = 4 activities)	112 (67%)	1.4	0.38	0.23	Transmission analysis	19 (11%)	0.63	0.56
					Response monitoring	53 (32%)	0.50	0.67
					Community consultation	29 (17%)	− 0.41	0.58
					Survey	30 (18%)	− 0.54	0.54
Other (specified)	–	–	–	–	Communications, coordination, infection prevention & control, collection & testing of specimens, reviewing of literature, treating cases, vector control	–	–	–

* Includes multiple answers per respondent

**Responder type (international or national responder)

### Communication

Respondents highlighted the importance of good communication skills necessary for implementing interventions, community engagement, as well as for team functioning: *“communication skills are key to successful public health interventions during a response. This goes for the community engagement side, but also within the team.”*

Respondents highlighted knowledge sharing as a challenge; commonly reporting a lack of recording of previous activities conducted by past deployees and poor handover. *“Not enough existing handovers (in a written format, efficiently written, not just hidden in the mission report of another team member) led to less efficiency in the team.”* One respondent claimed, *“existing staff acted as gatekeepers of knowledge, rather than centrally accessible information.”*

Almost 1:5 (32/166) of respondents reported that their deployment was too short for effective response (Table 2). The most common reported contract length was less than 1 month (32%, 52/163), with 55% (89/163) under 2 months (Table 1). One respondent reported, *“you barely figure out what you are doing before you go home”,* and another stated that *“it took time to develop relationships with colleagues until they were willing to allow me to have access to support the data management side of the work.”* The staff turnover also made it *“difficult to know 'who-was-who' both among international and local teams”.*

### Responder role

Understanding the role of the epidemiology emergency responder was a common challenge identified. Eighteen percent of participants reported that ‘*The team leader did not know how to use me/my skills’* (30/166), with significant difference noted between international and national responders (Table 2). The challenge of the team leader not knowing how to use the respondent was significantly different between respondents who had participated in three or fewer responses (20%, 20/79) compared to those who reported four or more (12%, 10/85) (p = 0.02).

Individual misunderstanding of role was claimed to be from to a lack of clarity in job descriptions, less than 50% (78/162) of respondents reported they had Terms of Reference (ToR) for the last response they had undertaken (Table 2). Of respondents with a ToR, 61% (48/78) of them stated they had a clear job description that matched the actual work they conducted (Table 1). “*My ToR were vague and misleading.”* The lack of role clarity was commonly identified as a team or organisational issue, *“the organisation I went out with was unsure what role they wanted to take within the larger epidemiological activities and as such my responsibilities were very unclear.”*

Respondents reported a common misunderstanding about the role difference between epidemiologists and medical professionals, with epidemiological work given to medical doctors. One respondent reported, *“medical staff don't understand the role of the epidemiologist and sometimes attempt to do the work of the epidemiologist, which can lead to some really bad/messy data.”* In times where epidemiologists conducted epidemiological work, there was a lack of understanding by the team leaders on why the work (such as surveillance system development) was important, *“[there were]. Leaders with no public health or epi[demiology]. background and [they]. could not appreciate the importance of setting up surveillance.”*

A significant difference was detected between responder types and those working in a ‘*cross-sectoral collaboration’* role (*p* = 0.004), with 23% (*n* = 15/66) international responders reporting this role compared to 43% (*n* = 41/96) of national responders (Table 3).

### Culture and relationships

**Fig. 1:**
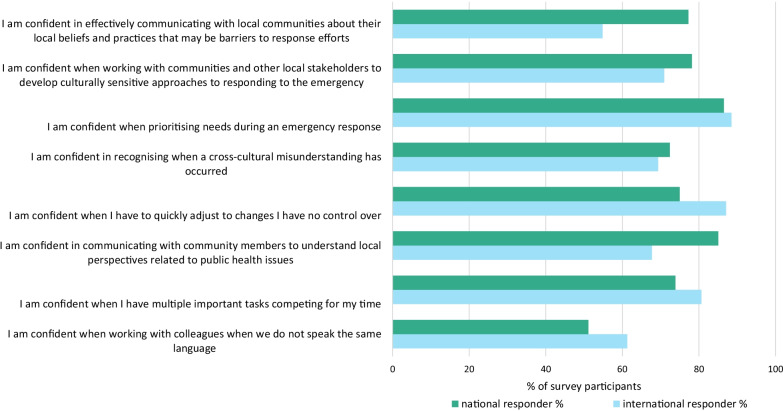
Comparison of international and national epidemiology emergency responders self-reported confidence in skills, 2019–2020

Few respondents identified items regarding *‘culture and relationship’* as a challenge (Table 2), and frequently self-rated their cross-cultural skills as strong (Fig. 1). Contrary to these results, free text comments frequently discussed challenges in relationship formation, communication, and cross-cultural challenges.

Survey participants acknowledged the importance of understanding culture to enable effective investigation and control measure design. *“My background is anthropology and I think it really helps when it comes to creating socially acceptable and impactful public health interventions”.* Some recommended cultural competence be included in briefings prior to deployment, while others discussed the importance of cultural mediators or community members in investigation teams; *“training on specific cultural difference could be offered per country/region before/during a deployment”* and *“this is too often overlooked and many US county-level epidemiologists lack cultural competence.”*

*“It is easier to respond to health emergencies in your country of origin than in another country (can speak the language, familiarity with cultural, political issues). International response demands more personal skills (cultural sensitivity) and need for institutional support from WHO or sending agency along with clear ToR and command structure.”*

### Technical skills

Participants reported few challenges regarding their technical skills (Table 2, Fig. 1); however, they recognised that technical knowledge alone was not enough to be effective during a response, *“it's not just a matter of subject matter experts having the academic knowledge. They must have the additional skills to be productive and not a burden in an emergency.”*

### Political dynamics and security

The three individual challenges grouped in ‘*political dynamics and security’* were the most listed challenges identified in the survey (Table 2). Navigating and understanding the political landscape was identified by 35% (58/166) of respondents, followed by the challenge of collaborating with external partners 27% (45/166), and 22% (32/166) identified security issues affected their capacity to work.. *“You learn very quickly that interagency coordination with courteous communications is the key to success”.* International responders reported challenges with understanding the political environment of emergency response (46%, 30/66), more than national responders (29%, 28/96) (*p* = 0.03) (Table 2).

Political issues identified from participant responses included: understanding the reasons behindisions, power struggles, corruption, and politics within and between teams. Respondents reported difficulties collaborating within and between teams across the response, with trust identified as a key issue.

Fifty-nine percent (63/107) of those who reported undertaking management roles identified *‘political dynamics’* as a challenge compared to 42% (*n* = 25/59) of non-management roles (*p* = 0.04). There was also a statistically significant difference between reporting ‘*political dynamics’* as a challenge and those who worked on ‘*cross-sectoral collaboration’* (*p* = 0.006). Sixty-eight percent (*n* = 38/56) of those who reported a cross-sectoral collaboration role identified political dynamics as a challenge compared to 45% (*n* = 50/110) of those who did not (Table 3).

### Language

When assessing self-confidence in skills, respondents reported the least confidence when working across languages (59%, 83/140 confident in this skill) (Fig. 1). When comparing insufficient language ability to responder type, we found a statistical difference (*p* = 0.001) with 23% (15/66) of international responders stating their language skills were insufficient to need compared to 5% (5/96) of national responders (Table 2). Respondents reported that there was a need for interpreters when working across languages and that responders who spoke the local language should be prioritised.

### Other challenges

Field experience was a common issue discussed by respondents. Respondents described a lack of support for new or inexperienced responders as well as the lack of emergency response entry-level positions. *“Most emergency deployments require you to be able to function autonomously as an 'expert' epidemiologist, which is difficult for someone starting their career”.* Others reported that emergency response was not an appropriate place to train professionals. *“Emergencies in remote locations or small countries often are not the right environment for support or professional development.”*

Respondents were concerned that post response evaluations were not occurring and that this limited the continuous improvement of responses. *“Post response evaluation is seldom conducted. The idea like AAR [after action review]. would be a good tool to regularly find out immediate issues and solve it to get prepared for the next event”.*

Additional challenges identified related to logistics and administration, such as access to funding, cars, accommodation, internet, phone, and food. *“Epidemiologists need to have some training related to resource management and basic understanding of finance and budgeting and costing of activities and plans”,* and *“plain old admin [administration]. support is necessary but not always provided.”*

### Support

Reporting on their most recent emergency response, 67% (103/153) of respondents stated they needed support to do their job well, 41% (64/156) reported they needed technical epidemiological support and 46% (72/156) non-technical support. Respondents commented on a need for the development of a pool of resources for use during emergency response and a contact list of experts available to answer questions.

Of those who stated they needed support, 77% (77/103) reported they had received support. There was no statistical difference between identified gender on whether there was a need for support during the response; however, there was a difference between support received and gender (*p* = 0.004), with more males reporting receiving support than females. There was a statistically significant difference in need of non-technical support between genders (*p* = 0.02) with 35% (30/85) of females reporting a need for non-technical support compared to 53% (42/79) of males.

In addition, we identified a statistically significant difference between national and international responders and reported support received, 81% (52/64) and 64% (25/39), respectively (*p* = 0.03).

## Discussion

The COVID-19 pandemic has exacerbated and bought to the fore the critical nature of a skilled, experienced, and well-trained public health workforce. Data for this study were collected prior to the COVID-19 pandemic, however, are more poignant now than ever. Our research key findings can be consolidated into three principal themes: leadership, politics, and communication.

### Leadership

#### Epidemiology workforce role

The need for local solutions to local problems is increasingly highlighted in the literature [3, 35–38]. Our survey found that international responders reported more challenges than national responders in navigating the political environment, collaboration, security, and language. We must begin to think wider than individual roles and reconsider the broader use and role of both local and international responders during emergency response and how they can more effectively interact.

Many of the challenges identified in our study stem from the structure of emergency response. Short deployments of international responders with limited experience and little or no knowledge of the local language, culture, or politics, hampers effectiveness. Our findings showed that national responders have ancillary strengths, such as a better understanding of the political environment, language, and culture, which may better support the identification of local needs and priorities [39–43].

The Global Preparedness Monitoring Board 2019 report indicated that emergency response systems need to better engage in community engagement during preparedness as well as response [7]. Local actors need to be at the centre of every response to ensure understanding of local context, history, cultural challenges [35, 37]. Increasingly, communities are demanding a leadership role within emergency response,[36]. and countries are enforcing restrictions on international workers to ensure country coordination and management [44]. It has been noted that although international responders deploy with good intentions, they can obstruct the work on the ground [44].

Just as the need for a rebalancing of power within the humanitarian aid sector between the Global North and South has been identified,[35]. discussions also need to be had within the public health emergency response sector. The inequity of response between the Global South and Global North during the COVID-19 response further accentuates why a review of emergency response framework is needed [45]. International emergency response stakeholders need to begin a global discussion ensuring adequate structures and frameworks for emergency response, and challenge the role of international responders to ensure leadership is centred at the local level to help address the complex challenges [3, 8]. Emergency workforce response needs to move towards a national structure, where international responders follow the lead of the national public health leadership and responders, and provide technical assistance to fill gaps and enhance capacity [3, 35, 39, 46].

This research identified the major role categories associated with the epidemiologist in emergency response. There is an urgent need to clarify epidemiological roles in the public health workforce in health systems internationally, not just during emergency responses. This study has identified that more work is needed to define roles by response type as well as to understand the minimum skills and experience needed to competently conduct each role, which in turn would inform the future field epidemiology and public health emergency response training.

Many of the challenges identified in this study are compounded by limited clarification or understanding of responder roles. Formalisation of required capacities and role of the medical and nursing professions during emergency response has been conducted; however, this has not yet been done for the epidemiology workforce [17]. Addressing clarity of roles for field epidemiologists will support effective recruitment, once required skills and experience are further refined. This would also lead to responders being more prepared for deployment and, therefore, increasing their effectiveness [18].

There is a need to sensitise management and the broader emergency response community on the role of the epidemiology workforce so the skills they bring to a response are better understood and productively applied. Training of Public Health leaders and managers has begun in many countries, to broaden the understanding amongst leaders and managers of the value of the epidemiology workforce. We recommend this sensitisation of leaders be conducted within emergency response teams and their collaborators.

### Support

While short-term deployment of international responders with limited experience continues, response teams need support mechanisms that ensure the short deployments are effective. Identification of the national responder needs during response could support more effective targeted international deployments and remote support mechanisms.

Although this research identified short international deployments as an issue affecting the effectiveness of response, it is important to note the prolonged response of COVID-19 has also raised issues of workforce exhaustion and burnout [47]. Workforce support and management is essential to protecting individuals from harm as well as ensuring an uninterrupted and effective response. In early 2021, the WHO Director-General stated that to strengthen health security, the global health emergency workforce needs investment and strengthening at all levels [48]. Although all levels do need strengthening, the primary focus should be to ensure local workforce support during emergencies. When national systems are overwhelmed, international emergency response needs to be appropriate to need, with a focus on strengthening and supporting the national responder. Longer term measures include addressing human resource issues and team structure to ensure collective competence during emergency responses [16]. This includes training and upskilling of the new and current workforce, accreditation of the epidemiology profession, clarification of roles, defining minimum skills and experience needed for roles, and providing a support system to assist responders while working on an emergency [16]. Revision of the Incident Management System (IMS) approach to response with details on the epidemiological role during emergency response would support this strengthening.

Earlier research into the training gaps identified key areas for workforce support programs such as FETPs to focus on strengthening local workforces [2]. Other workforce support research has identified mentorship-type support helps to mitigate inexperience of the response workforce especially when navigating complex political environments [49, 50].

### Politics

#### Required skills

The COVID-19 pandemic has clearly demonstrated that large public health events require responders with specialist skills and expertise to appropriately address the crisis. The pandemic response has also exposed the political nature of outbreaks and the critical role of politics in defining response direction andision-making. [3, 19, 20]. Public health specialists and epidemiologists are critical to inform evidence-basedision making; however, the Independent Panel report criticised political leadership that either failed to hear or act on this expertise to prevent SARS-CoV-2 transmission and guide control [19]. Our study has highlighted that epidemiology responders often do not understand the political dynamics of an outbreak, or find them difficult to navigate. Politics is a central component of outbreak management, and we need to better equip epidemiologists with political intelligence through future epidemiology training [38, 49, 51].

#### Gender

Although our respondents were equally distributed across identified gender, we identified gender-based differences in who received needed support, with males receiving more support. In this increasingly female-dominated field, we need to do better to support women, especially when they request support. These findings are congruent with research and reports identifying gender biases in leadership and gender representation in global health [3, 8, 52–54].

#### Responder type

When we compared the challenges and roles of national to international responders, we found that national responders were less likely to identify politics as a challenge and they were more likely to engage in cross-sectoral collaborative work. The importance of, and the need for, local based response is well documented in the literature [40, 55–60]. Future outbreaks need to embrace this to ensure a cross-sectoral approach is taken to emergency response emergency, and collaboration rather than siloed work is essential.

### Communication

Our research shows a broad range of communication challenges. In a recent study of epidemiology training needs, communication skill development was identified as in need of strengthening [2]. To improve the effectiveness of the epidemiology workforce during emergency response, communication strategies need to be developed. The COVID-19 pandemic has emphasised the need for clear communication, and prompt sharing of resources, information and knowledge. [8, 61, 62]. Improving communication skills of the epidemiology workforce would also support use of evidence to inform the response [2]. Defining and communicating the role of the epidemiologist toision-makers and emergency responders would also help the broader response community to realise how the epidemiology workforce could be better integrated and utilised during a response [8, 16].

### Limitations

Early consultations with emergency response organisations identified the absence of comprehensive emergency response workforce databases. This meant it was not possible to conduct representative sampling of this population. Given this, care must be taken when applying lessons learnt from this paper to the broader applied epidemiology workforce. To lessen the impact of selection bias, we used multiple pathways to recruit participants. As this study included participants representing all WHO regions, we made the survey available in French and English to increase representation. There were varying timeframes between the most recent emergency response and the time of survey completion, potentially leading to different levels of recall. A time lag between responses and completion of the survey may have been advantageous as the individual had time to reflect on their role and the challenges they experienced [63, 64]. This survey was based on self-reporting of challenges as perceived by the individual and may not be representative of the applied epidemiology workforce. Finally, this survey was conducted prior to the COVID-19 pandemic and does not specifically capture the current pandemic challenges.

## Conclusions

Leadership, politics, and communication skills need strengthening to ensure the public health workforce can effectively manage health security. Many of the challenges identified in this research stem from the current structure of global emergency response. International emergency response stakeholders must begin a global conversation to reconsider the use and role of national and international responders during emergency response as the risk of another crisis similar or more severe than COVID-19 is plausible. The public health epidemiology workforce needs to be adequately and effectively prepared.

## Data Availability

All data relevant to this manuscript are included within the manuscript tables, figurers and/or reference links.

## Abbreviations

AAR: After action review
COVID-19: Corona Virus Infectious Disease 2019
FETP: Field Epidemiology Training Programme
IHR: International Health Regulations
IMS: Incident Management System
JEE: Joint External Evaluation
REDCap: (Research Electronic Data Capture)
TEPHINET: Training Programs in Epidemiology and Public Health Interventions Network
ToR: Terms of Reference
WHO: World Health Organization
